# Urinary cotinine cut-off value in categorising cigarette smoking status in healthy pregnant women at term

**DOI:** 10.2478/aiht-2025-76-4052

**Published:** 2025-12-30

**Authors:** Nataša Brajenović, Irena Brčić Karačonji, Andreja Jurič, Jelena Kovačić, Sandra Stasenko, Tatjana Mioč, Iva Miškulin, Lana Škrgatić, Martina Piasek, Jasna Jurasović

**Affiliations:** Institute for Medical Research and Occupational Health, Zagreb, Croatia; University of Rijeka Faculty of Health Studies,Croatia; Merkur University Hospital, Zagreb, Croatia; Poliklinika Harni, Zagreb, Croatia; University Hospital Centre Zagreb, Zagreb, Croatia

**Keywords:** biomonitoring, creatinine-adjusted cotinine, pregnancy, second-hand smoke, smoking validation, biološki monitoring, kotinin preračunat na kreatinin, pasivno pušenje, trudnoća, validacija pušenja

## Abstract

Future mothers tend to underreport intentional or unintentional tobacco smoke exposure to avoid social stigma, and urine levels of cotinine, the major metabolite of nicotine can serve as a valuable biomarker of tobacco smoke exposure to resolve such uncertainties. When establishing the cut-off level to discern non-smokers from active smokers, however, one should bear in mind the shorter half-life of cotinine in pregnant than non-pregnant women to avoid misclassification of maternal smoking status. The aim of our study was to determine the urinary cotinine cut-off level to objectively detect active smokers and to see if any participant characteristics were associated with underreporting. To do that, we collected spot urine samples from pregnant women with normal vaginal delivery at term, self-reported as non-smokers (n=123) or smokers (n=33), in the maternity ward before delivery. We also took their sociodemographic, cigarette smoking, and clinical data, as well as clinical data on their newborns using a questionnaire. To ensure objective classification of participants by smoking status, cotinine levels were quantified in urine using gas chromatography-mass spectrometry. The receiver operating characteristic (ROC) curve analysis showed that the optimal cut-off value of urinary cotinine to discern pregnant non-smokers from smokers at term were 120 µg/L or 144 µg/g creatinine, yielding 94 % sensitivity and 96 % or 97 % specificity. Respective to these cut-off values, 4.1 % and 3.3 % of self-reported non-smokers were classified as current smokers. Our findings support the use of urinary cotinine, whenever feasible, to reduce reporting bias in pregnancy studies, and, given the altered nicotine metabolism during pregnancy, future epidemiological studies should bear in mind that urinary cotinine cut-off values may depend on the stage of pregnancy.

## Abstract (hr)

Kotinin, kao glavni metabolit nikotina u mokraći, prepoznat je kao vrijedan biološki pokazatelj izloženosti duhanskome dimu kako bi se prevladala nesigurnost samoprocjene koja nastaje nenamjerno ili zbog negativnih društvenih konotacija povezanih s pušenjem budućih majki. Treba uzeti u obzir kraći poluživot kotinina u trudnica, u usporedbi sa ženama koje nisu trudne pri određivanju granične vrijednosti kako bi se razlikovale nepušačice od aktivnih pušačica i izbjegla pogrešna klasifikacija pušačkoga statusa budućih majki. Uzorci mokraće zdravih trudnica s normalnim ročnim porođajem koje su se u upitniku izjasnile kao nepušačice (n=123) ili pušačice (n=33) prikupljeni su u rodilištu prije porođaja. Podaci o sociodemografskim karakteristikama, navici pušenja cigareta i klinički podaci o majkama i njihovoj novorođenčadi zabilježeni su u upitniku. Kako bi se osigurala objektivna klasifikacija ispitanica prema pušačkome statusu, razine kotinina kvantificirane su u mokraći tehnikom plinske kromatografije spregnute sa spektrometrijom masa. Analiza krivulje osjetljivosti dijagnostičkog testa (ROC) pokazala je da su optimalne granične vrijednosti kotinina u mokraći za razlikovanje rodilja nepušačica od pušačica 120 µg/L i 144 µg/g kreatinina, s osjetljivosti 94 % i specifičnosti 96 % odnosno 97 %. Prema tim graničnim vrijednostima, nepreračunato ili preračunato na kreatinin u mokraći, 4,1 % odnosno 3,3 % onih koje su se izjasnile kao nepušačice klasificirano je kao trenutne pušačice. Izračunata granična vrijednost za kotinin u mokraći osigurala je točniju kategorizaciju pušačkih navika među rodiljama u usporedbi s podacima dobivenima samoprocjenom. Naši rezultati podupiru potrebu određivanja kotinina u mokraći, kada god je to moguće, sa svrhom smanjenja nesigurnosti samoprocjene pušačkog statusa ispitanika. U budućim epidemiološkim istraživanjima treba voditi računa o vremenu uzorkovanja s obzirom na promjene u metabolizmu nikotina tijekom različitih stadija trudnoće koje imaju ulogu u odabiru granične vrijednosti tog biološkog pokazatelja.

Health risks associated with cigarette smoking in women include a myriad of disorders, ranging from effects on maternal reproductive health and postpartum disorders to chronic diseases and malignancies. In pregnancy, smoking is linked to placental and foetal membrane disorders as well as health risks in the offspring that can be manifested as a number of conditions soon after birth and/or later in adult life ( 1,2,3,4,5,6,7). According to a meta-analysis by Lange et al. (8), the estimated prevalence of smoking during pregnancy in Europe was 8.1 %, with 30.6 % of daily smokers continuing to smoke throughout pregnancy. In Croatia, this prevalence exceeds the European average with some of the highest reported rates before and during pregnancy (9). A recent Eurostat report for 2019 confirmed Croatia as one of the EU countries with the highest smoking prevalence among women aged >15 years and ranking second among women aged 15–29, of whom 26.6 % reported smoking daily or occasionally. Back then, fewer than 1 % women used smokeless tobacco products (10).

Smoking status is considered one of the key covariates in the studies evaluating the impact of environmental exposures on offspring during the entire reproductive period of women. However, pregnant women often underreport tobacco use due to avoid criticism from the society and health professionals. Previous data indicates that up to 35 % of pregnant smokers falsely declare themselves as non-smokers (11), which renders doubtful self-reported data in epidemiological studies. To overcome this uncertainty, cotinine as the major metabolite of nicotine in urine, has widely been used as a reliable, non-invasive biomarker of tobacco smoke exposure (6, 12,13,14,15,16). Although biochemical verification of the smoking status has its limitations (e.g., inability to confirm long-term abstinence, higher cost, and implementation challenges), it is particularly valuable in populations where underreporting is expected. Importantly, the half-life of cotinine is much shorter in pregnant (8.8 h) than in non-pregnant women (16.6 h) due to increased clearance and accelerated metabolism (17), which complicates the interpretation of urinary cotinine levels in epidemiological studies on pregnant women and argues against applying cut-offs established for the general population. Another issue is the use of different analytical techniques to determine urinary cotinine. Although immunoassays are fast and relatively cheap, they are less specific than chromatographic analyses and may overestimate urinary cotinine levels (18). As no consensus has yet been achieved regarding an optimal urinary cotinine cut-off value to distinguish smoking from non-smoking pregnant women, this may lead to discrepancies in interpreting research results of the effects of maternal environmental exposure on child health (13, 19). Although several urinary cotinine cut-off values have been proposed so far, none concerns gas-chromatography measurements in pregnant women at term.

In our recent research project “Assessment of Daily Exposure to Metals and Maternal Individual Susceptibility as Factors of Developmental Origins of Health and Disease” (METALORIGINS, 2018–2022), we assessed environmental exposure to nutritionally essential and main toxic metals in women during child-bearing age, considering that diet and tobacco smoking are the key sources of metal exposure in general population. Human biomonitoring was employed to evaluate maternal exposure to metals in mother-child pairs and its effects on the child. Therefore, it was crucial to collect as accurate data as possible on maternal smoking history before and during pregnancy. The aim of this study, as an important part of the above project, was to determine the urinary cotinine cut-off level to objectively discern active smokers from non-smokers at term and to identify self-reported characteristics associated with participants who misreported their smoking status.

## PARTICIPANTS AND METHODS

### Study participants and questionnaire

Questionnaire data and biological samples were collected between January 2018 and February 2019 from 156 healthy postpartum mother-infant pairs with normal vaginal delivery at term, recruited in the maternity wards of two clinical hospitals in Zagreb, Croatia, as described in earlier reports (19, 20). Study participants who met inclusion criteria were asked to complete a questionnaire to collect relevant data regarding their general health and obstetrics/gynaecology history, sociodemographic characteristics, and personal habits focused on tobacco use and cigarette smoke exposure. All gave written informed consent before their enrolment. The study protocol was approved by the ethics committees of three participating institutions in Zagreb, Croatia: Merkur University Hospital (No. 03/1-5102/1), University Hospital Centre Zagreb (No. 021-1/43-18), and the Institute for Medical Research and Occupational Health (No. 100-21/17-07).

The participants were initially asked to classify themselves as a non-smoker, former smoker, or current smoker and to provide detailed information on their smoking behaviour during pregnancy (whether they smoked during the entire pregnancy, the number of cigarettes smoked per day after learning to be pregnant, and timing and reasons for smoking cessation during pregnancy). They were also asked at what age they started smoking, how many cigarettes a day they had smoked before pregnancy, when they quit smoking (for former smokers), and whether they were exposed to secondhand smoke (SHS). This last set included questions about the smoking habits of their household members and estimated duration of exposure at home, at the workplace, and in public places.

Following a detailed review of responses (to resolve contradictory answers between initial self-classification and smoking behaviour reported in more detail in additional answers), the participants were categorised in two groups: active smokers or non-smokers. Active smokers (n=33) were those who reported smoking during entire pregnancy. The non-smokers category comprised both never-smokers and former smokers who had quit before or early in pregnancy.

### Urinary cotinine as a biomarker of cigarette smoke exposure

Urine samples were collected from the participants into polypropylene screw-cap containers (Sarstedt, Nümbrecht, Germany) in the maternity wards before delivery, stored at 4 °C, and immediately transported to the analytical laboratory, where they were stored at −20 °C until analysis.

Cotinine was extracted from urine using headspace solid phase microextraction (HS-SPME), and its mass concentration was determined using gas chromatography-mass spectrometry (GC-MS) following the method described by Brčić Karačonji et al. (21). Briefly, cotinine from 1 mL of urine sample was extracted with an 85 µm polyacrylate SPME fibre (Supelco, Bellefonte, PA, USA) and analysed on a Varian 3400 CX gas chromatograph coupled with Saturn 4D ion trap mass spectrometer (Varian, Palo Alto, CA, USA) in the electron impact (EI) mode. The method’s limit of cotinine detection (LOD) was 0.25 µg/L. Urinary creatinine was measured with a spectrophotometric method based on Jaffe’s reaction (22).

### Statistical analysis

Numerical variables (e.g. maternal age, cotinine levels) were presented by median (min–max or IQ25–IQ75). Differences between groups were tested with the Mann-Whitney *U*-test and Fisher’s exact test for continuous and categorical variables, respectively. Associations between the variables were tested with Spearman’s correlation coefficients. For statistical calculations, undetectable cotinine concentrations were set at 0.5×LOD. The significance was set at p<0.05.

Urinary cotinine cut-off values, with and without creatinine correction, were estimated using the receiver operating characteristic (ROC) curve analysis. Statistical analyses were run on Statistica version 14.0.0.15 (TIBCO Software Inc., Palo Alto, CA, USA) and R software, version 4.5.2 (R Foundation, Vienna, Austria).

## RESULTS

Table 1 shows the general characteristic of the study participants, their self-reported smoking habits, and urinary cotinine concentrations. Smokers were younger and had significantly lower education level than non-smokers. Pre-pregnancy BMI did not differ between the groups. Among smokers, cigarette consumption was relatively low, with most (91%) reporting smoking up to 10 cigarettes per day.

**Table 1:** General characteristics and urinary cotinine values in pregnant women at term grouped by self-reported smoking habits

	**Non-smokers (n=123)**	**Smokers (n=33)**	**p**
Age (years)	33 (21–44)	29 (21–38)	**<0.001**
Education^a^			**<0.001**
Primary	7 (5.7 %)	3 (9.1 %)	
Secondary	47 (38.2 %)	24 (72.7 %)	
Tertiary	69 (56.1 %)	6 (18.2 %)	
Pre-pregnancy BMI	22.9 (14.7–36.3)	23.0 (16.0–36.6)	0.469
Former smokers	52 (42.2 %)	/	
Number of cigarettes smoked per day during pregnancy	/	7 (1–20)	
Urinary cotinine (µg/L)	2.30 [<LOD–6.70]	1034 [611–2668]	**<0.001**
Urinary cotinine (µg/g creatinine)	2.06 [<LOD–9.55]	1305 [420–2096]	**<0.001**

Data are presented as medians and ranges, (min–max)or [IQ25–IQ75], or as a number and percentage (%). Differences between the groups were tested with the Mann-Whitney *U*-test or Fisher’s exact test for continuous or categorical variables, respectively. Significant differences (p<0.05) are marked in bold.

a Education level was categorised using the International Standard Classification of Education (ISCED): primary (ISCED 0–2), secondary (ISCED 3–4), and tertiary (ISCED 5–6). LOD – limit of detection; BMI –body mass index

Urinary cotinine concentrations were below the LOD in 29 % of the samples. Among non-smokers, 28.5 % reported SHS exposure (Table 2). SHS-exposed women were younger and had lower education level (p<0.05). More than half of SHS-exposed non-smokers (51.4 %) were former smokers. Significantly higher urinary cotinine concentrations in participants who reported SHS exposure seems to confirm their report.

**Table 2:** Second-hand smoke (SHS) exposure and urinary cotinine in non-smoking pregnant women at term

	**Non-smokers reporting no exposure to SHS (n=82)**	**Non-smokers reporting exposure to SHS (n=35)**	**p**
Age (years)	34 (21–44)	31 (21–37)	**0.002**
Education^a^			**0.017**
Primary	2 (2.4%)	3 (8.6%)	
Secondary	26(31.7%)	18 (51.4%)	
Tertiary	54 (65.9%)	14 (40.0%)	
Urinary cotinine (μg/L)	1.00 [<LOD–4.20]	6.50 [1.40–23.8]	**<0.001**
Urinary cotinine (µg/g creatinine)	1.08 [<LOD–4.09]	7.05 [1.48–22.2]	**<0.001**

Results are presented as medians and ranges, (min–max) or [IQ25–IQ75], or as the number and percentage (%). Differences between the groups were tested with the Mann-Whitney *U*-test or Fisher’s exact test for continuous or categorical variables, respectively. Significant differences (p<0.05) are marked in bold.

a Education level was categorised using the International Standard Classification of Education (ISCED): primary (ISCED 0–2), secondary (ISCED 3–4), and tertiary (ISCED 5–6). LOD – limit of detection

**Figure 1:**
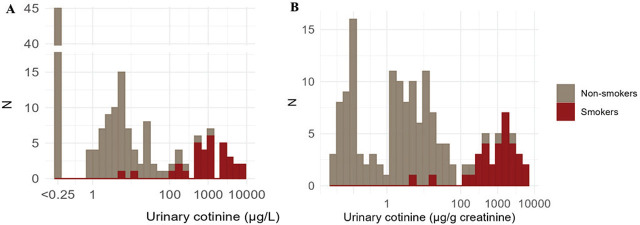
Distribution of (log-scaled) urinary cotinine concentrations in pregnant women at term (n=156) grouped by self-reported smoking habits and expressed as A) µg/L and B) µg/g creatinine

**Figure 2:**
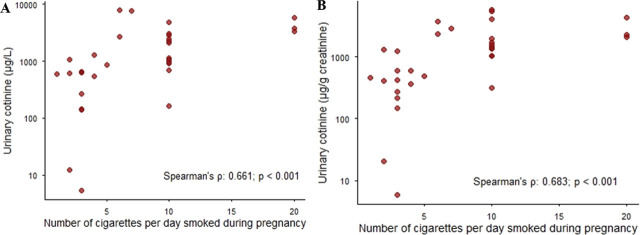
Association between the number of cigarettes smoked per day and (log-scaled) urinary cotinine concentrations in participants who reported smoking during pregnancy (n=33), expressed as A) µg/L and B) µg/g creatinine

Figure 1 shows the distribution of urinary cotinine concentrations in our study group, while Figure 2 shows a significant association between urinary cotinine (either corrected for creatinine or not) with the number of cigarettes smoked during pregnancy. Spearman’s correlation between urinary cotinine levels with and without creatinine correction was high (r=0.980; p<0.001).

**Figure 3:**
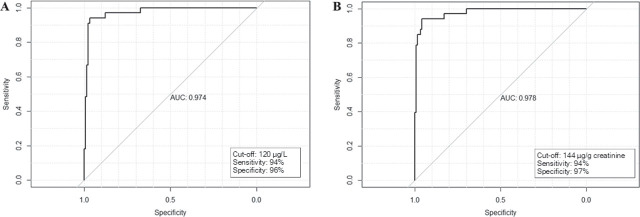
Receiver operating characteristic (ROC) curves describing cut-off values for urinary cotinine, expressed as A) µg/L and B) µg/g creatinine, that distinguish non-smoking from smoking healthy pregnant women at term

ROC curve analysis identified 120 µg/L and 144 µg/g creatinine as the optimal cut-off urinary cotinine levels to distinguish pregnant non-smokers from smokers at term (Figure 3). The cut-off level above of >120 µg/L helped to identify five of the 123 self-declared non-smokers (4.1 %) as smokers and the cut-off level of >144 µg/g creatinine four of them (3.3 %). Conversely, two participants who declared themselves as smokers had values below 120 µg/L or 144 µg/g creatinine (Table 3).

**Table 3:** Misclassification rates of self-reported smoking status with urinary cotinine concentrations based on the identified urinary cotinine cut-off values (120 µg/L and 144 µg/g creatinine) in pregnant women at term

	**Non-smokers with cotinine <120 µg/L (n=120)**	**Smokers with cotinine >120 µg/L (n=36)**	**Non-smokers with cotinine <144 µg/g creatinine (n=121)**	**Smokers with cotinine >144 µg/g creatinine (n=35)**
Ratio to self-reported				
Non-smokers	118/123 (95.9 %)	5/123 (4.1 %)	119/123 (96.7 %)	4/123 (3.3 %)
Smokers	2/33 (6.1 %)	31/33 (93.9 %)	2/33 (6.1 %)	31/33 (93.9 %)
Urinary cotinine (μg/L)	2.15 [<LOD–5.95]	994 [577–2562]	2.20 [<LOD–6.00]	1034 [598–2668]
Urinary cotinine (µg/g creatinine)	2.02 [<LOD–6.88]	1315 [440–2194]	2.03 [<LOD–7.05]	1324 [460–2292]

Results are presented as the number and percentage (%) or medians and ranges [IQ25–IQ75]. LOD – limit of detection

## DISCUSSION

This study is the first to validate self-reported smoking among pregnant women in Croatia using urinary cotinine cut-off values of 120 µg/L and 144 µg/g creatinine to distinguish smokers from non-smokers at term. These thresholds demonstrated high sensitivity and specificity and helped to identify participants as either smokers or non-smokers, who would have remained misclassified based on self-reported questionnaire data alone. Misclassification among non-smokers (3–4 %) falls within the 3–6 % range previously reported in pregnant populations (18, 23, 24), reinforcing the added value of biochemical verification.

Accordingly, whenever possible, tobacco smoke exposure should be objectively verified with biomarkers such as nicotine in hair or cotinine in urine, plasma, or saliva (1, 16, 25). Among those, urinary cotinine is a reliable marker of recent tobacco smoke exposure and suitable for large-scale studies (6, 12,13,14,15, 23, 25), but it reflects total nicotine exposure, irrespective of the source. At the time of our study (2018), the use of newer tobacco and nicotine products, such as e-cigarettes, heated tobacco products, smokeless tobacco, and nicotine patches, was quite rare in Croatia (10). In the meantime, alternative nicotine-delivery systems have become widely available, and future studies relying on urinary cotinine may increasingly capture nicotine exposure from sources other than conventional cigarette smoke (27, 28). In addition to tobacco and nicotine products, trace amounts of nicotine can also be found in certain foods of the *Solanaceae* family (e.g., tomatoes, potatoes, aubergines/eggplant), but their concentrations are extremely low and unlikely to affect urinary cotinine findings.

Consistent with earlier studies using the enzyme-linked immunosorbent assays (ELISA), liquid chromatography-mass spectrometry (LC-MS), or GC-MS (13, 14), urinary cotinine concentrations in our study were markedly higher among smokers than non-smokers (approximately 500 times) as well as among the participants reporting exposure to SHS than those not reporting it (approximately 7 times higher). Our findings that younger participants with lower education prevailed among both active and passive smokers corroborate earlier reports on pregnant women (29, 30). Nearly 30 % of non-smokers reported SHS exposure, and more than half of these women were former smokers, suggesting that lifestyle patterns may contribute to continued environmental exposure even after smoking cessation. Although urinary cotinine levels in SHS-exposed women were markedly lower than those of active smokers, they confirmed measurable passive exposure, which is still relevant given the established associations between prenatal SHS exposure and possible adverse birth and child health outcomes (31, 32).

Comparable data on smoking habits, SHS exposure, and validation of self-reported smoking status with urinary cotinine are missing in Croatian vulnerable population groups, but recent median GC-MS urinary cotinine measurements in a pilot study in young Croatian adults (mean age 24) (26) were higher in non-smokers (35.36 µg/L) and lower in smokers (54.44 µg/L) than those observed in our study population.

A persistent challenge in the field is the absence of a universally accepted urinary cotinine cut-off value to classify smoking status during pregnancy, further complicated by pregnancy-related physiological changes. During pregnancy, nicotine metabolism increases due to elevated activity of CYP2A6 and UGT2B10, accelerating nicotine biotransformation and cotinine clearance (17, 25, 33). As a result, cut-off values and biomarker thresholds established in non-pregnant populations may underestimate true tobacco exposure during pregnancy. In our previous study (34), we observed a decrease in hair nicotine concentrations from the first to the third trimester in non-smokers, passive smokers, and active smokers, even though cigarette consumption remained largely unchanged, which further supports the need for pregnancy-specific exposure thresholds. However, available evidence regarding changes in urinary cotinine across trimesters is inconclusive. While one study (24) reports lower levels in the third than second trimester, and another (35) lower levels in the second than the first or third trimester, the large-scale data from the Japan Environment and Children’s Study (JECS) cohort (23) show no significant variation by gestational week. These mixed findings likely reflect both behavioural changes during pregnancy and physiological adaptations that may influence cotinine concentrations across gestation.

Currently, no consensus has been reached regarding the cut-off value for urinary cotinine in pregnant women, although several thresholds have been proposed obtained with different analytical techniques. Generally, urinary cotinine cut-off of 100 µg/g creatinine is considered an acceptable threshold to distinguish smokers and non-smokers (36, 37), but most proposed cut-off values by a number of studies range between 20 and 300 µg/L (5, 12, 23, 24, 29, 36, 38,39,40). Our cut-off values are comparable to those reported in settings with higher cigarette smoke exposure, including SHS, and baseline cotinine levels, which have been shown to accurately differentiate between smokers and non-smokers (5, 12, 40). However, the small sample size in our study, especially that of smokers, increases the uncertainty regarding the selection of optimal cut-off values. For example, the cut-off value for unadjusted cotinine (120 µg/L) was obtained as a midpoint between non-smoker with cotinine of 101.1 µg/L and smoker with cotinine of 139.5 µg/L. As there were no participants with a cotinine level between these two values (101.1–139.5 µg/L), setting the cut-off value to any level in this range would be equally supported by our data, i.e., it would have resulted in the same sensitivity, specificity, and, consequently, misclassification rates. Similarly, equally plausible values for creatinine-adjusted cut-offs based on our data distribution would be in the range of 139.7–144 µg/g creatinine.

Our analysis of misreported cases provides further insights. Among women who declared themselves as non-smokers, three (i.e., two when urinary cotinine levels were adjusted for creatinine) were former smokers, which suggests a more likely scenario of reduced or intermittent cigarette consumption than quitting. In the remaining two misreported cases, we found implausibly high urinary cotinine (>1300 µg/g creatinine). Questionnaire analysis showed that these two participants, who declared themselves as never-smokers, had the lowest educational level, which is in line with earlier findings linking lower education and socio-economic status with falsely denying smoking behaviour (29, 30). Therefore, education seems a potential determinant of misclassification. As for the two self-reported smokers who had cotinine concentrations below the cut-off, we believe that this points to inconsistent smoking behaviour or prolonged abstinence before sampling.

Although urinary cotinine levels with and without creatinine adjustment correlated significantly between themselves as well as with the reported cigarette consumption, we support creatinine adjustment in similar future studies due to convincing evidence that adjustment of urinary cotinine for creatinine concentration improves the correlation between urine and plasma cotinine (16). It is also worth emphasising that analytical techniques used for cotinine analysis and creatinine correction have a pivotal role in making decision on cut-off value that will minimise misclassification rate.

This study has several limitations. We did not ask the participants for information on active and passive exposure to tobacco smoke 24 h before sampling (or established the timing of the last cigarette or SHS exposure), which may have contributed to exposure misclassification. Also, we did not exclude self-reported non-smokers with implausibly high cotinine levels to avoid artificially lowering the misclassification estimate, but their exclusion from ROC analysis could have further refined the cut-off estimate. Finally, the study was conducted in a single urban region and may not have captured exposure patterns in other parts of Croatia.

In summary, this study identified urinary cotinine cut-off values for distinguishing active smokers from non-smokers among healthy pregnant women at term in Croatia. These thresholds demonstrated high sensitivity and specificity (94 %) and identified misreporting participants, which confirms the added value of biochemical verification to self-reporting. Given the altered nicotine metabolism during pregnancy, future epidemiological studies of smoking in pregnant women should bear in mind that urinary cotinine cut-off values may depend on the timing of urine sampling, that is, on whether the samples are taken during the first, second, and/or third trimester of pregnancy, as well as at term. Our findings support the use of urinary cotinine, whenever feasible, to reduce reporting bias in epidemiological pregnancy studies.
